# *In vitro* Models of the Small Intestine for Studying Intestinal Diseases

**DOI:** 10.3389/fmicb.2021.767038

**Published:** 2022-01-04

**Authors:** Sang-Myung Jung, Seonghun Kim

**Affiliations:** ^1^Jeonbuk Branch Institute, Korea Research Institute of Bioscience and Biotechnology (KRIBB), Jeongeup, South Korea; ^2^Department of Biosystems and Bioengineering, KRIBB School of Biotechnology, University of Science and Technology (UST), Daejeon, South Korea

**Keywords:** small intestine, *in vitro* model, *ex vivo* model, 3D culture, disease model, intestinal glycans, host-microbiome interaction

## Abstract

The small intestine is a digestive organ that has a complex and dynamic ecosystem, which is vulnerable to the risk of pathogen infections and disorders or imbalances. Many studies have focused attention on intestinal mechanisms, such as host–microbiome interactions and pathways, which are associated with its healthy and diseased conditions. This review highlights the intestine models currently used for simulating such normal and diseased states. We introduce the typical models used to simulate the intestine along with its cell composition, structure, cellular functions, and external environment and review the current state of the art for *in vitro* cell-based models of the small intestine system to replace animal models, including *ex vivo*, 2D culture, organoid, lab-on-a-chip, and 3D culture models. These models are described in terms of their structure, composition, and co-culture availability with microbiomes. Furthermore, we discuss the potential application for the aforementioned techniques to these *in vitro* models. The review concludes with a summary of intestine models from the viewpoint of current techniques as well as their main features, highlighting potential future developments and applications.

## Introduction

The small intestine is a key part of the digestive system that has critical roles essential for sustaining life. It plays crucial roles in food digestion and nutrient absorption, as well as homeostasis maintenance *via* host–microbe interactions. The small intestine has a long tubular structure of 6 m–7 m in length and an inner diameter of 3 cm–4 cm. The inner side of the small intestine, called the lumen, is an epithelial cell layer whose microstructure consists of villi and the basal crypt. Moreover, the small intestine has a large surface area around 250 m^2^; thus, its vast microstructure area enhances the efficient absorption of a wide range of smaller molecules that result from the digestion of macromolecules in the stomach, i.e., amino acids from proteins, sugars from polysaccharides, glycerol, and short-chain fatty acids from lipids, etc. (Biernat et al., 1999; Sokolis and Sassani, 2013). Gut intestines are complex ecosystems under anaerobic conditions that include a variety of microorganisms and are rich in nutrients. Such human gut microbiota are relevant to human health and pathogenesis (Tuddenham and Sears, 2015; Hillman et al., 2017; Shortt et al., 2018).

Gut microbiomes predominantly consist of bacterial genera, including *Faecalibacterium*, *Roseburia*, and *Bifidobacterium*, even though other main groups of microorganisms, such as archaea, fungi, protozoa, and viruses, can be observed (Bédard et al., 2020). These microbiome consortia can uptake or metabolize exogenous dietary substrates in the gut and convert them into valuable metabolites; notable examples are bile acids, short-chain fatty acids, branched amino acids, trimethylamine N-oxide, tryptophan, and indole derivatives. These microorganisms can also utilize endogenous host compounds, mucins, and other glyco-conjugates derived from gut intestines.

Mucins are O-linked glycan-attached glycoproteins secreted from goblet cells and anchored to the intestinal epithelial layer. Heavily glycosylated mucins have unique properties, such as viscoelasticity. The gel-forming mucin glycan covers the whole intestinal lumen in a thick mucus layer. They mask the host epithelial cell layers and block pathogenic microorganisms that are potentially responsible infection. Thus, these glycan layers protect epithelial cells from harsh gut conditions as well as many infectious microorganisms. On the other hand, these branched glycoconjugates can also facilitate niches for gut microbiomes. Glycoconjugates on the host cell surfaces are used by a variety of pathogenic microorganisms as infectious mediators for attachment and invasion. Furthermore, bacterial glycosidases and glycosyltransferases can modify the host surface glycans, leading to pathogenicity. Therefore, several types of O-glycan-decorated mucins in gut intestines are considered to be cause of glycan-mediated infection resulting from interactions between the host and microbiomes (Ziegler et al., 2016).

**Figure 1:**
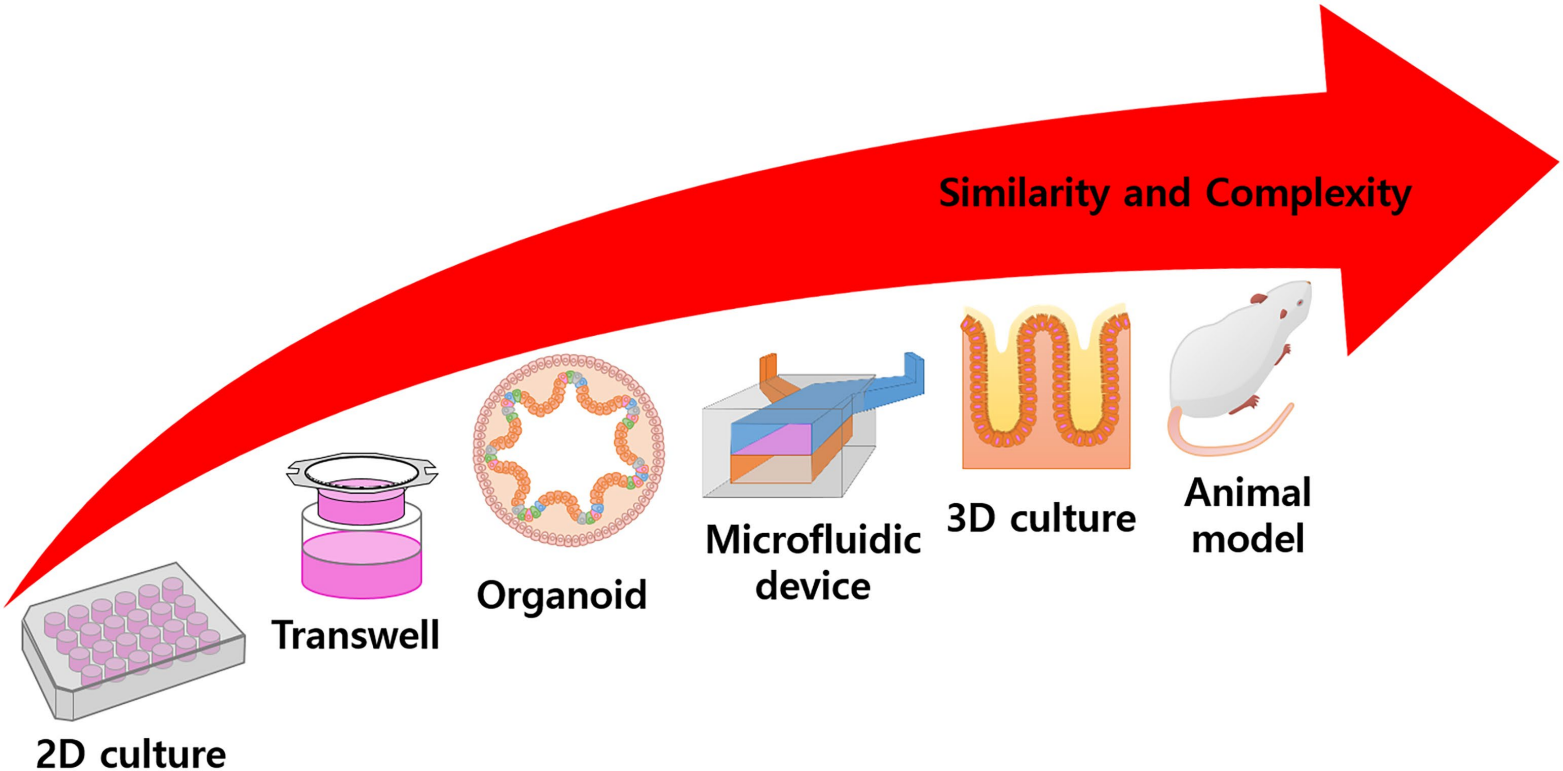
Overall scheme of experimental model. Conventional 2D culturing is rather simple and has high productivity, but application is limited to target treatment experiments. In the Transwell system, the culture area is floated into the media and can simulate mass transfer or other activities. Many models try to simulate specific functions or structures using complex techniques, such as organoid differentiation, microfluidic devices, 3D scaffold fabrication, tissue engineering, etc. In animal models, the body represents a complete model, but there are problems with observation and ethical issues. In general, with increased complexity, the similarity of the model also increases.

Gut microbiota, in both humans and animals; interact directly with the host by the production of a diverse reservoir of metabolites obtained from exogenous or endogenous substances. Examining intestine homeostasis between the gut microbiota and the host immunity is a key factor in assessing the health status of a body. Moreover, the role of gut microbiota in immune homeostasis and autoimmunity has been intensively studied to evaluate the interaction of microbial communities and the host immune system for understanding pathogenesis and related diseases as well as for developing novel immuno- or microbe-based therapies. However, various exogenous/endogenous conditions would be influent to dramatically alter the profiles of microbes and their metabolites, making their impact on host health status change. Therefore, the interactions between the hosts’ cells and microbiomes have been studied using *in vitro* and *ex vivo* models instead of animal models. Nowadays, *in vitro* or *ex vivo* intestinal models are established and utilized to evaluate host–microbiome interactions. In this review, we describe the current emerging technologies of *in vitro* and *ex vivo* intestinal models adopted in place of animal intestinal models (Figure 1). We also discuss their benefits and the future perspectives for the development of gut-mimetic models for bacteria–gut epithelium interactions.

## Roles and Characteristics of the Intestine

The gastrointestinal tract (also referred to as the GI tract, GIT, digestive tract, digestion tract, and alimentary canal) extends from the mouth to the anus, with the intestine being the long, continuous tubular organ responsible for digestion and absorption. It mainly comprises the small and large intestine, where each is divided into three parts, according to their main roles and structures, as duodenum, jejunum, and ileum (Lopez et al., 2020).

Starting at the duodenum and ending at the ileum, dietary substrates are digested by enzymes to obtain nutrients. Proteins are digested by trypsin, chymotrypsin, and additional enzymes to obtain amino acids. Fat is emulsified and transformed into micelles by bile salts and lecithin. Most nutrients are absorbed from the small intestine, which has a large surface area, then transferred to blood vessels and delivered to the liver or other organs. Subsequently, undigested and unabsorbed residues are transferred to the large intestine.

As described above, the intestine is an organ designed to digest food and absorb digested residues, including nutrients and moisture. The intestine is the largest organ in the body, almost 6 m–7 m in length in adults, and is tightly packed into the abdominal cavity. The length plays a role in maximizing the residual time for the whole volume of digested residue passing through the intestine to facilitate the complete absorption of nutrients and moisture. Moreover, the intestine is its ripples, consisting of villi and crypts, which maximize the surface area to increase the absorption efficiency in a limited volume. Crypts are the indented parts of the ripples, composed of stem cells and transit amplifying cells, while the villi are the protruding parts, composed of differentiated cells, including enterocytes, goblet cells, and endocrine cells. In addition, the microvilli are high-density, small hair-like cellular structures on the villi, which increase the surface area (Biernat et al., 1999; Sokolis and Sassani, 2013).

The intestine interacts directly or indirectly with gut microbiota, which can metabolize digested substances catalyzed by secreted bacterial enzymes. These intestinal microbiota and their metabolites can influence to host metabolism through the regulation of various cellular mechanisms in the organ (Martin et al., 2019). On the other hand, for the homeostasis and maintenance of intestinal tissue, a defense system is needed to prevent pathogenicity *via* microbial invasion. Harmful external pathogens are primarily sterilized when passing through the stomach. Nevertheless, the intestinal tissue would be still at risk for infection due to potential pathogens present in the gut organs. To overcome risk, intestinal tissues produce self-defensive substances called mucins, glycoproteins, for the defense system against infectious diseases.

**Figure 2:**
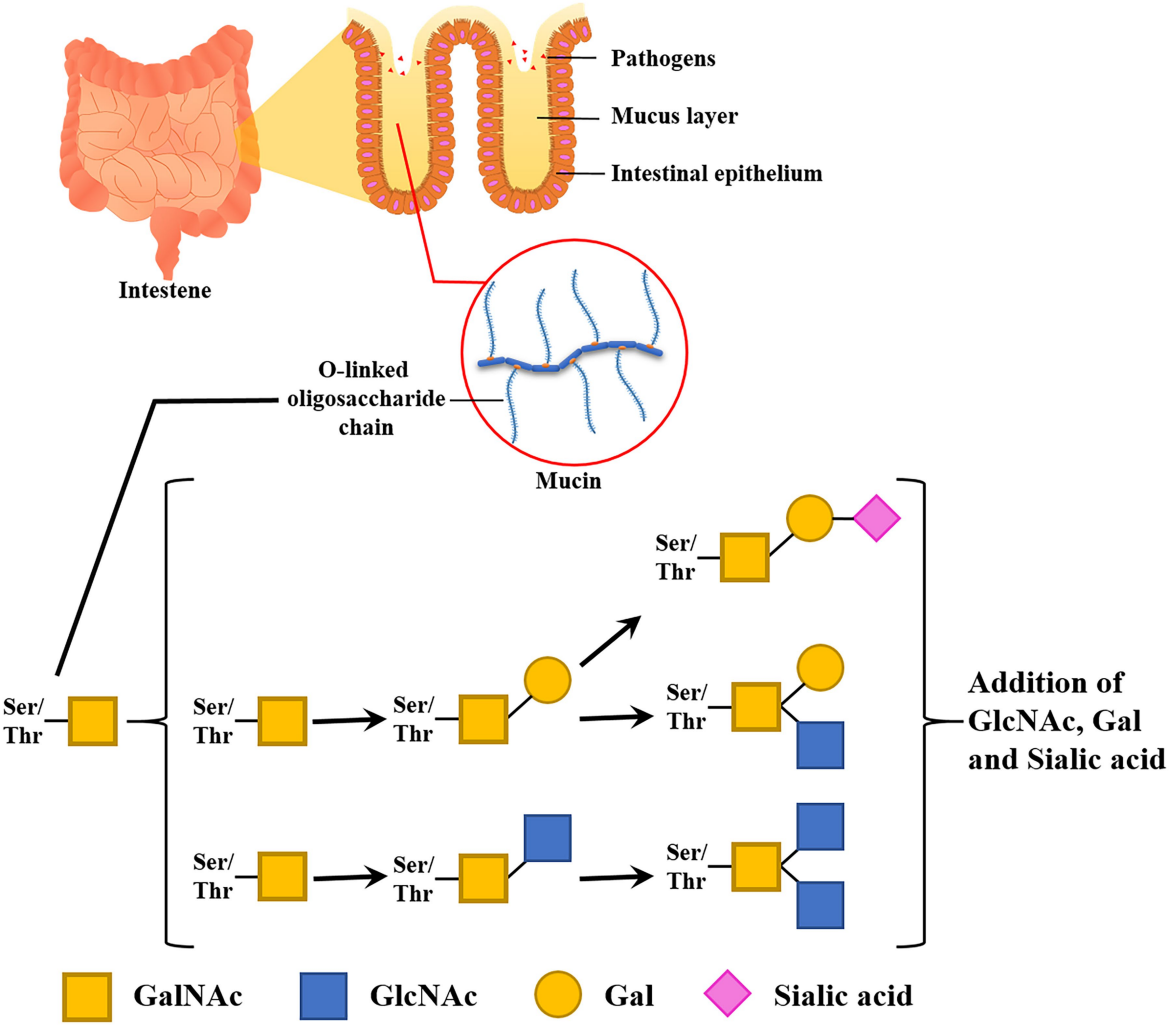
Functions and compositions of intestinal glycan (mucin). A mucus layer covers the intestinal lumen. This thick layer prevents the epithelial cell layer from invading microbes. Glycan entraps pathogens and infectious microbes in its dense O-linked oligosaccharide chain.

Goblet cells sparsely located among enterocytes secret mucus composed of many different molecules, with mucins forming the basic skeleton (Birchenough et al., 2015; Johansson and Hansson, 2016). Mucin consists of a group of transmembrane and gel-forming proteins with high O-glycosylation. The glycosylation consists of O-linked oligosaccharides (glycans), including mainly N-acetylgalactosamine (GalNAc), N-acetylglucosamine (GlcNAc), galactose (Gal), fucose (Fuc), and a terminal sugar sialic acid (Sia). The polypeptide backbones for mucin have a serine or threonine residue and N-acetylgalactosamine attached to the residue for initiating O-linked oligosaccharide elongation. These O-linked glycoconjugates stretch out in high density and have strong viscoelastic properties (Figure 2).

Mucin forms a gel-like structure and covers the entire surface of the gastrointestinal tract. More than 20 types of mucins have been observed. Among them, MUC2, MUC5AC, MUC5B, and MUC6 are secreted form for the mucus layer of the intestine. Mucin anchors to the cell surface, is secreted into the lumen, or is taken up through ingestion to sustain its levels and status. Therefore, susceptibility to infection or pathogen invasion depends on the presence of the glycan anchoring site, where pathogens will adhere. Thus, glycans can reduce infection susceptibility by acting as receptor decoys, because they are in contact with but not directly attached to the epithelial tissue. This is one of the main characteristics of the epithelial tissue of the intestine and lung, and the presence of mucin not only secures the robustness of the epithelial tissue but also directly affects the mass transfer (Devine and McKenzie, 1992; Linden et al., 2008; Grondin et al., 2020).

## Types of Intestine Models

### *In vivo* Model

There are several ways in which the intestinal environment status can be altered abnormal for the construction of a disease; this is a physiological approach that is used in conjunction with other methods. While rodent models have been especially important in promoting a mechanistic understanding of human diseases, there are cases where it is scientifically appropriate to use large animals. Various species are used for *in vivo* models, such as murine, other rodents, canids, swine, and monkeys, and each has its own advantageous features as a model (Hugenholtz and de Vos, 2018). Among those species, rodents, especially murine rodents, represent an important species for intestinal modeling, as the human intestine microstructure is almost completed replicated in the rodent intestine, and achieving this degree of replication is difficult using cell cultures.

Even though there are advantages to using animal models, they do not fully simulate the human intestine and there are risks involved. Rodents have similar microbial species to the human intestinal ecosystem, but the proportions of those species are vastly different. For this reason, even though the rodent model is valuable as a primary preclinical model, a direct comparison to the interaction or disease relationships between the intestinal microbiome and intestinal tissues in humans is problematic. The differences between animals and humans are also apparent when considering other aspects; for example, the ecosystem differs between humans and rodents not only in terms of the population but also the composition of the digestive residue, because most digestive residues are used as metabolites of the microbiome and are converted to small molecules or bioactive molecules, such as short-chain fatty acids, glycerol, etc. Therefore, direct comparisons between humans and animal models are not necessarily valid, and an alternative model that can effectively control these factors is required (Xiao et al., 2015; Hugenholtz and de Vos, 2018).

The animal model is a traditional and conventional platform that is used when biological data are needed from specific organs in complex environments. As a model, it has a complete network and structure; however, methods for preparing animal models require more concentration and a longer preparatory period than for *in vitro* models is required to complete and maintain a specific status prior to experiments, as mentioned above.

Models of the intestine are essential for research on enteric pathogens. The mechanism of interaction between foodborne pathogens, mammalian hosts, and intestinal microflora remain largely unknown, including the mechanism of microbial attachment and crosstalk with the host epithelium and the preventive and curative effects of probiotic bacteria.

Various experimental models have been developed for clinical studies and animal models have typically been employed. In those models, germ-free mice were widely used as an *in vivo* model experimental system to understand the underlying mechanisms and estimate human mechanisms.

Although the traditional animal model offers a good standard model for biomedical studies, including studies on cellular signaling pathways, potential drug candidates, and the design of drugs for probiotic healthcare and infectious disease, it has obvious disadvantages. First, the intestines of animals cannot completely mimic the human intestine. For example, Cao et al. (2006) found that a rat model could not completely simulate the drug metabolism and oral bioavailability of humans based on differences in the underlying molecular mechanisms (Stappaerts et al., 2015). Large deviations were observed in the animal models, and it was challenging to reproduce the obtained results. Therefore, it was difficult to determine the relevance of clinical data and any physiological results in the host (Clancy, 2003; Suntharalingam et al., 2006).

There is also criticism among scientists that mechanistic approaches between animal and human models are insufficient. Not only are there differences in metabolism, but physiological differences also occur in incomplete simulations with animal models. For example, some pathogens or viruses only infect certain species or induce symptoms, which is an especially serious limitation for an intestinal model being applied in host–microbiome research. Given these differences, it is not always possible to create a suitable model for the intestinal pathogens and gut environment of humans. The gut microbiome is a complex microbial group, and each species has a unique profile, which applies not only to common and benign microorganisms but also gut pathogens, which can vary in terms of microbiome or species. For example, the gut bacterium *Listeria monocytogenes* cannot infect rodent species, due to metabolic differences between humans and rodents. When fully simulating *L. monocytogenes* infection, the microbe should mediate cell extrusion of the enterocyte. For cell extrusion, the adherens junction of the enterocyte is targeted by *L. monocytogenes*, with InlA/E-cadherin playing a role in *L. monocytogenes* transcytosis. However, the interaction between InlA and E-cadherin of the non-permissive hosts (i.e., mouse and rat) is not sufficient to simulate the interaction in humans, which is a kind of permissive hosts. It would be necessary to modify transgenic mice with humanized InlA to fully simulate listeriosis in humans including InlA-mediated transcytosis or translocation (Drolia et al., 2018; Drolia and Bhunia, 2019). Therefore, rodent intestinal models are not suitable for studying the infection of listeriosis fully, which is a serious infectious disease in humans. Even though there is another model using oral infection of mice and it showed that *L. monocytogenes* expressing murinized InlA (InlAm) with a high affinity for E-cadherin, but it excludes InlA-mediated transcytosis or translocation. It is a weakness of animal model that it needs additional modifications, and verifications to replace humans. Additionally, in many cases, these animal models show a part of whole mechanisms. In this sense, an organism is an aggregate of complex networks and systems and, if even a single phenomenon occurs differently, it is hard to predict the result and analyze it mechanistically using other species. Indeed, many studies have identified several biological responses that are specific to humans and cannot be simulated in models of other animal (Shanks et al., 2009).

Further, it is expensive and time-consuming to obtain reliable results regarding human responses and physiology using *in vivo* models from other animals. For the development of *in vivo* models, sufficient space and many facilities and materials are needed for breeding experimental animals. An animal is a complete organism with complex networks and systems that maintains its own homeostasis. For this reason, it is impossible to induce an immediate change in its condition for experiments. Ideally, the animal’s condition is gradually changed, and limited methods are available for this, which typically involve diet regulation and compound injection over time to finalize a specific animal model. The intestine, in particular, is the organ with the most complex ecosystem and is directly affected by diet. Another method is to use transgenic animal models, but few verified models exist, and they are usually more expensive than wild-type animals. The swine intestine has remarkably close resemblance to that of humans and the results from this model demonstrate good relatability to human intestine. Based on these advantages, swine models are very widely used and considered high quality; however, the animals are too large and take up too much dedicated time to be used in the laboratory. Finally, there are concerns and debates about animal welfare, and the promotion of animal models is not in accordance with the trend of minimizing animal use for research purposes. With the EU leading, many societies are eager to reduce animal experimentation in all biotechnological industries, such as cosmetics, pharmaceuticals, and healthcare (Lunney, 2007).

To overcome the above problems and enhance experimental availability, various models have been developed, including *ex vivo* and *in vitro* models. With *ex vivo* models, we can transfer the complexity of *in vivo* tissue to the laboratory. Researchers can use the complete intestinal structure and cell composition based on intestinal tissue segments. Human tissue is harder to secure than animal tissue, but the advantage is that the model can fully simulate human intestine *in vivo*, and researchers can make feasible predictions for clinical trials using the results. For example, *ex vivo* models are widely used in pharmacological studies on the transport of drugs across intestinal barriers, gastrointestinal hormones, and metabolism (Ripken and Hendriks, 2015). *In vitro* intestine models could potentially solve the problems of animal models, namely the dissimilarity between humans and animals, the burden as a model for laboratory use and ethical issues. First of all, *in vitro* models have good human predictive power; most *in vitro* intestine models are composed of human cell lines, which mean the model can satisfactorily simulate *in vivo* responses. The mechanistic approaches are sufficient to yield data that support the interpretation of the results for *in vivo* states (Cencic and Langerholc, 2010). In addition, *in vitro* models are easier for researchers to use than animal models. Fewer facilities and less equipment are required for cell cultures and they are well standardized for repeating experiments to get reliable data. They can be developed for ready availability and easy handling in high-throughput testing. This is a strong advantage for a model system in the discovery of drug candidates and pathways of signaling and metabolism.

### *In vitro* Model

As already mentioned, the conventional animal intestine model has a few limitations. First of all, it is hard to achieve homogeneity of experimental units (environments and other requirements) because each individual has congenital characteristics, derived from complex networks and ecosystems of the animal. These networks and ecosystems help to achieve valuable results from preclinical trials; on the other hand, these complex elements are intricately related to each other, making it difficult to focus clearly on the target in the experiment (Lu et al., 2014; Ziegler et al., 2016). Therefore, to ensure data reliability, more replications are required for animal experiments than *in vitro* experiments. In particular, as the intestine has the most complex ecosystem in an animal’s body, it is hard to perform direct observation, highlighting the limitations. In addition, the cost, time, and ethical issues of animal experiments are always a concern (Knecht et al., 2020; Pilla and Suchodolski, 2020).

**Figure 3:**
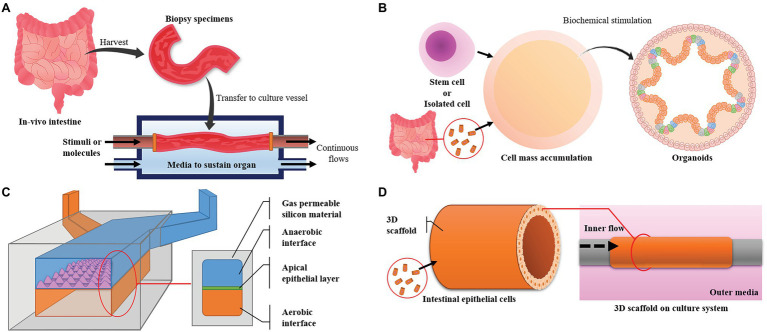
Major intestinal models and their designs. **(A)** The *ex vivo* model uses intestine harvested from experimental animals and maintains its live state. It has high similarity, but the live state is hard to maintain. **(B)** Organoids are derived from pluripotent stem cells or cells harvested from *in vivo* tissue. The model has high similarity in function and cell composition but can only be maintained for a limited time. **(C)** Microfluidic devices can control the environment, are easy to observe and make it easy to focus on targets but have low productivity and a small area for experiments. **(D)** Three-dimensional (3D) cultures can provide large areas for experiments and high productivity but advanced techniques are required to simulate *in vivo* conditions and maintain a high level of uniformity.

Due to these limitations, there has been demand for the development of *in vitro* models. Cell-based models are in the spotlight as an alternative to animal models based on their numerous advantages, including that they allow target-restricted experimentation, direct observation, and continuous analysis. In the case of general *in vitro* experiments, deviations and noise in the experimental results can be minimized, because researchers design the experiments with only the specific elements they want to check. Such well-controlled and restricted experiments can be used to derive more detailed and accurate research results. The various intestinal models are introduced at Figure 3 and Table 1.

**Table 1:** Types of intestine models and their properties.

Type	Origin	Simulating degree (complexity)	Configuration	Uniformity	Methods	Viability	Applications	Features
*Ex vivo*	Intestinal tissue from animal	Complete structure, with muscular layer, all component cell types	Epithelial cells, enteroendocrine goblet cells, Paneth cells, blood and lymph vessels, M cells, Peyer’s patches, and immune cells	Large deviation among individuals	Intestinal rings, intestinal segments, and everted sac, using system	Under 2 h (over 10 days when using biopsy only)	Regional absorption mechanisms, GI hormone release, and drug transport	Relatively simple, already formed and available for harvest, and hard to maintain
*In vitro*	Cell line	Low simulation [two-dimensional (2D) culture]	Epithelial cells, immune cells	Low deviation	Cell culture on culture plate	Continuous culture	GI hormone release, transcriptomics, and early immune response	Easy to configure, limited to simulating cell components, structure hard to simulate
3D culture	Cell line	Simulated cell components (co-culture), structure (scaffold), and dimension (size)	Epithelial cells, enteroendocrine goblet cells, and immune cells	Low deviation	Cell culture on Transwell, 3D scaffolds	Continuous culture	Regional absorption mechanisms, transcriptomics, and drug transport	Simulate structure using scaffolds, ability to separate areas
Organoids	Isolated crypt cells, stem cells	Simulated cell components (raw cell from tissue, cell differentiation), parts of lumen and villi	Epithelial cells, enteroendocrine goblet cells, Paneth cells, M cells, Peyer’s patches, and immune cells	Large deviation, hard to control	Digested crypt tissue and re-suspension, stem cell differentiation	Continuous culture	GI hormone release, transcriptomics, and early immune response	Requires special skill, simulates detailed structures but not large structures

## *Ex vivo* Intestine Model For Direct Experimentation

The *ex vivo* model is a transitional model between *in vivo* and *in vitro* models. The model consists of whole intestine or specific tissues and a designed culture system to prolong its survival and activity. This model is used to obtain selective advantages from both *in vivo* and *in vitro* models. The advantages from *in vivo* models are that it has full cell type composition; suborgans made up of groups of specific cells (glands, vessels, etc.) and complete tissue structure and ecosystem. Direct observation and simple treatment or stimulation of tissues are the advantages of *in vitro* models. The intestinal tissue has a tubular structure and apical cell layer, so the inner and outer environment can be separately controlled. Researchers can circulate and control the inner fluid and its flow to understand the interaction between tissue and microbiome (Westerhout et al., 2015; Pearce et al., 2018).

However, this model requires high-level techniques for successful culture and prolonged intestinal function. The intestine is exposed to contaminants during the extraction process and many microorganisms, including infectious pathogens, are also contained in the lumen. In the case of an *ex vivo* model using animal tissue, another disadvantage is that there are differences in anatomical structure and physiological conditions, including diet and microbiome, derived from differences among species. The disadvantages complicate the extrapolation of data to humans. In addition, the *ex vivo* model does not avoid ethical issues, because the organs and tissue are extracted from animals (Pearce et al., 2018).

## Two-Dimensional Culture Model For Simple and Fast Screening

The two-dimensional (2D) model is a basic cell-based model of culturing a single cell or multiple cells on a flat surface. To create an intestinal model, cell lines, typically Caco-2, HT-29, T-84, IEC, and other enterocytes, are generally used to construct an apical epithelial layer, and additional cell lines are used to add specific functions to the model, such as mucus secretion and immune response. The cells are attached at the bottom of the culture vessel, which is then filled with a medium on the cell layer such that it is easy to initiate treatment, i.e., with a molecule and continuously observe the system. In this way, the model is simple and easy to construct and conduct experiment with, so it can be used similarly to a conventional model in screening assays (Simon-Assmann et al., 2007; Smetanová et al., 2011).

However, 2D cultures are not suitable for simulating intestinal structure. The culture surface of the model is usually a flat plastic vessel bottom, so it is hard to change the shape. In addition, this intestinal model can be used only in limited situations for interactions between tissue layers and microorganisms. There is only one vessel for the culture medium, so it is impossible to treat living microorganisms, which have higher growth rates than inoculated animal cells, because microorganisms exhaust the nutrients of the medium and secrete various harmful substances into animal cells, such as proteolytic enzymes, pathogens, etc. For this reason, microorganisms are treated for only a few hours or are inactivated by fixing to prevent overgrowth (Co et al., 2019). This 2D model has a rather simple structure and configuration, so it is easy to use and the model composition can easily be modified, including changing the cell line and medium component. Because of these advantages, *in vitro* 2D models are widely used, and various models have been established and are currently being used to screen the pharmacology and toxicology of new drug candidates.

## Three-Dimensional Culture Model With Transwell To Simulate *in vivo* Structure

The Transwell® system is a useful piece of equipment with separate reservoirs for supplying nutrients and treatment substances. The porous membrane housed in the Transwell insert is located in the middle of the culture vessel, and the vessel is divided into two reservoirs by the membrane. Therefore, it is suitable for constructing an apical cell layer such as the intestinal epithelial layer (e.g., intestinal lumen and blood vessel). The Transwell model has the additional advantage of easily simulating the intestinal structure. There is a porous membrane in the Transwell, so cells can be organized into homogeneous layers. From these layers, it is easy to organize models using the Transwell with high standardization, but some characteristics or properties of the original organ or tissue need to be simplified. Typically, it is optimized to construct cellular monolayers and homogeneous environments. However, *in vivo* tissues are composed of three-dimensional (3D) cellular multilayers and exist in heterogeneous or complex environments suitable for each part of the tissue. In general, the Transwell intestine model is used to study mass transfer and barrier functions through cellular layers because it offers the possibility to use on-target screening and construct a high-throughput system by mass production, even if there are some limitations in the representation (Ghaffarian and Muro, 2013; Srinivasan et al., 2015).

To overcome these limitations, the membrane can be modified to mimic the intestinal microstructure (especially crypts and villi). For effective 3D culture on the membrane, several ECM-like substrates (e.g., collagen, hyaluronic acid, hydrogel, or Matrigel®) are often embedded with the cell line to construct the basal cell layer. These substrates are made of a substance composed of a tissue basement layer and have properties that represent multi-cell layers by ensuring area-to-cell dispersion. From the cell dispersion and multi-cell layer formation, cell-to-cell interaction is induced and cellular function is activated in single cells; finally, this helps cells to be organized into tissues and affects the tissue robustness and adaptation to external environments. The basement layer provides an adherent residue to cells and a viscoelastic property to the tissue, finally helping to organize the tissue into a specific structure. If a designed scaffold is used to replace the Transwell membrane, the villus and crypt architecture of the epithelial layer is arranged before cell inoculation, enhancing the level of simulation of the whole model by biofabrication (Sung et al., 2011).

Except for structural development by basal layer construction, the heterogeneous and complex environment is mimicked in a modified model. First, the different kinds of cells to be inoculated are increased in the model. In the simple composition of cells, only those with a single phenotype are inoculated to organize the epithelial layer, such as enterocyte-like Caco-2 or another cell line derived from intestinal epithelium. After this simple model, additional cells are added to enhance the model’s degree of simulation. Mucin-secreting cells are a typical additional cell line, such as goblet cells or goblet cell-like cells. The mucin layer is effective in mass transfer tests in the intestinal epithelial model and helps to represent physiological features of *in vivo* intestinal tissue. To more closely mimic cell type diversity, stem cells or precultured organoids are inoculated in the Transwell (Costa and Ahluwalia, 2019).

In order to focus more on physiological and immunological causes, immune cells are used as candidates for additional cell lines. When using the Transwell, immune cells are cultured in a chamber on the side opposite to the apical cell layer. The immune cells, typically dendritic cells, are dispersed into the chamber or cell layer and migrate depending on the immune response. When using this Transwell model, pathogen candidates, microbiome, and various intestinal substrates are treated to assess the susceptibility to infection and stimulation of the immune system. To investigate host–microbe interactions in the advanced model, not only cells of human origin but also microorganisms are used for co-culture in the apical chamber. A semipermeable membrane that mimics the intestinal epithelial layer separates the reservoir, and microbes are inoculated at the apical chamber, thus resulting in an *in vitro* representation of the complex *in vivo* ecosystem.

Apart from cell type diversity, the external environment of the *in vivo* intestine can be simulated by supplying various supplements. ECM components can be supplemented to support the basal layer, such as certain proteoglycans and fibrous proteins (collagen, elastin, fibronectin, and laminin). When stem cells are cultured, growth factors, complements, and small molecules for the niche are supplemented to control differentiation and maturation (Sung et al., 2011). This type of advanced Transwell model was developed to overcome the excessive simplification and reductionism of earlier models, resulting in a more complex system that may recapitulate *in vivo* physiology more accurately but with disadvantages in terms of cost, culturing difficulty and reproducibility.

## Lab-On-A-Chip For Simulating Intestinal Ecosystem

The purpose of the lab-on-a-chip, a type of microfluidic device, is to simulate the target tissue as closely as possible within a limited area; it was developed to achieve simulation at micro- and nanoscales. The microfluidic device contains a hollow channel less than 1 mm wide with a continuously perfused flow. The microchannel limits the volume to a microliter and supports fine control of the fluid from nano- to microliter scales. With the use of a peristaltic pump, the culture medium is perfused at a constant flow rate into the microwidth channel, forming a laminar flow in the channel, which helps in easily estimating and controlling the fluid hydrodynamics. The microstructure is printed using a fabricated template and a silicon material such as PDMS; it is easy to produce using simple protocols and with secure gas permeability for cell cultures. The cells and media are added in the structure to form a model. It is easy to control the culture environment in the microfluidic device because the volume of the culture is small, and the structure is accordingly designed (Vickerman et al., 2008; Song et al., 2020). In addition, this model can be designed by the researcher using silicon material, i.e., the model has been modified for the advantages of TEER measurement and visualization (Henry et al., 2017).

In the intestine model, this device simulates the intestine microstructure. The channel of the device is used as the lumen of the intestine and the medium flow into the channel is used to represent intestinal flow. This flow rate can be regulated to simulate intestinal flow in the lumen or shear stress on the cell surface located at the intestinal epithelial layer, so the channel can be inhabited by cells arranged to simulate physiological features of a tissue or the whole organ. In the intestinal model developed using a microfluidic device, two chambers are constructed in the device and two air conditions are set in each chamber. In one chamber, intestinal residue is simulated with microorganisms or without microorganisms and just containing metabolites, while, in the other, an environment for animal cells that supplies the proper culture conditions, such as media, gas, etc., is realized. Finally, a cell culture layer is placed between the two chambers to simulate the intestinal structure (Giusti et al., 2014).

Even though there are many advantages, the microfluidic chip is too small and fine and can only deal with a small volume, so it is hard to produce a chip that can be stable and provide fine control during the whole culture period. A microfluidic chip of human intestine has a more complex structure and conditions in terms of intestine-specific microstructure (villi and crypts), so the challenges are more apparent in the model. To overcome these challenges, computer techniques are widely applied in microchip fabrication (e.g., soft lithography) and lumen flow control. This fine control is a typical advantage of the microfluidic chip to manipulate luminal components, such as microbes, nutrients, drugs, or toxins. In response to these challenges, the aim of various studies has been to enhance the complexity of the intestinal simulating microfluidic chip by focusing on the production of mucin, the unidirectional flow, and the cocultivation of mammalian and microbial cells without contamination (Workman et al., 2017; Bein et al., 2018). Accordingly, several designs of the microfluidic chip have been ideated, such as two-channel, *ex-vivo*, multichannel, and gut chip.

Recently, the complexity of microfluidic chips simulating the intestine has increased with the application of tissue engineering techniques. The model has been developed in the direction of including additional neighboring channels for surrounding environments, such as microvessels, immune cells, and pathogenic substrates. In some studies, these subchannels are used to generate mechanical stress in the cell culture chamber and are applied to mimic peristaltic deformations of the *in vivo* intestine using peristaltic pumps. Recently, Don Ingber’s group focused on reproducing intestinal characteristics on a microfluidic chip using the two-channel microfluidic chip, which has two channels parallel to the porous membrane. The intestinal epithelial structure was constructed on the membrane. Based on this model, they developed additional elements, such as the mechanical intensity to applied to the model, cocultivation of mammalian and microbial cells, etc. (Kim et al., 2016).

Based on those studies, the technologies and designs of microfluidic organ-on-a-chip models have undergone dramatic development, making this the most industrialized type of intestinal model. This model is used in scientific discovery, preclinical evaluation, and safety assays. There are many suppliers for these high-simulated microfluidic devices known as microphysiological systems (MPSs). However, many pharmaceutical companies have been worried about the availability and reliability of MPSs compared to conventional proven processes. Recently, there has been a sharp increase in MPS examples, including novel designs and concepts in the literature. Following those studies, companies have been increasing their production of the model slowly but steadily. Among the major suppliers are Emulate, MIMETAS, TissueUse, Wyss, and AIST (Marx et al., 2020).

As mentioned above, the advantages of the microfluidic device are its very high degree of simulation rate and uniformity. Recently, a model was developed to expand on its capabilities with additional functions to mimic the *in vivo* intestine. However, the size of the model is extremely limited, and it is not easy to increase its productivity, so there are limits to its use as an assay model. For these reasons, most of the past microfluidic intestine models had their own standards in individual academic labs, so their results and reliability may be too varied to share and collect as big data. These limitations of the model may be resolved by recently emerged companies that will commercialize the technology through scale-up manufacturing. Nonetheless, trials must be conducted before the microfluidic intestine model becomes a tool commonly used in academic and industrial research laboratories.

## Organoid

Organoids are cellular aggregates with a spherical shape that include aggregates of stem cells and cell groups with diverse cell types extracted from tissues. Stem cell-derived organoids are formed by embryonic or pluripotent stem cells and construct spheroid-like cell masses. After cell mass formation, various supplements, including complement and signaling molecules for cellular niches, induce the differentiation and maturation of stem cells into intestinal tissues to generate similar tissues. Cell-extracted organoids are recovered from *in vivo* intestinal tissue and single cells are obtained from the tissue through enzymatic digestion. Organoids are formed by inoculating the obtained cells into ECM components, Matrigel®, or a researcher-designed scaffold. The general form of the organoids is a spherical cell mass, and the features of intestinal enterocytes are expressed at the inner wall of the spheroid. An empty space (lumen) is created in the center in a form surrounding the cell layer. The advantage is that intestinal organoids, through self-organization, represent typical features of *in vivo* tissue, such as 3D structures and microstructures. A highly curved epithelium structure is self-organized by crypts and villi, similar to in the *in vivo* intestinal epithelium. Organoids are organized with a central hollow region with a curved structure, similarly to the intestinal lumen. The apex site of a crypt consists of Lgr5+ stem cells and Paneth cells, while the central region of an organoid consists of differentiated cells, not stem cells (Nakamura and Sato, 2017).

The lumen is completely separated from the outside by the cell layer, so it is used to configure and maintain an anaerobic state. At this point, several researchers have constructed an anaerobic state at the lumen and treated microbes by syringe injection to simulate a highly similar intestinal environment (Simian and Bissell, 2017). The advantages of these organoids are that they have high reproducibility of intestinal tissue and can be used to observe interactions with microorganisms, but the model configuration is difficult and the uniformity is poor for repeated experiments, which may cause problems with the reliability of repeated experimental results. In addition, since the size of the cell mass continues to increase according to the culture period of the organoid, there is a limit to how long the structure can be maintained. It is difficult to maintain a constant steady state because the shape and environment change depending on the culture time (Panek et al., 2018). Specifically, self-organization is an advantage of organoids, but quality cannot be controlled by the organoids since they are always organized heterogeneously in terms of shape.

Furthermore, because each organoid forms a closed lumen when cultured within the surrounding ECM gel and the cellular layer of the organoid and the surrounding gel intersects, it can be difficult to sample or manipulate the experimental components (e.g., microbes, nutrients, drugs, or toxins) into the internal lumen. Furthermore, it is hard to mimic the *in vivo* biomechanical stress and lumen flow. For these reasons, the structure of the organoid model also significantly limits its availability to study many critical intestinal functions (e.g., mass transfer, absorption, drug metabolism, or microbiome interaction). Despite these disadvantages and limitations, organoids show fast growth in applications spanning from assays to regenerative medicine. The potential of structural self-organization and cell differentiation make the model available as a “mini-gut,” and attempts have been made to engraft them in mice for testing.

## Three-Dimensional Intestine Models in Tissue Engineering

The models mentioned above simulate the intestinal ecosystem and particular tissue structure. The models are limited in size to increase the simulation degree, so they have disadvantages in terms of narrow observation and producibility. To overcome these disadvantages, typical features of the *in vivo* intestine are selected for simulation in the tissue-engineered model, namely microstructure, whole structure, and lumen environments. First, the structure is simulated using a tubular scaffold, microstructure is added using additional scaffold or basement substances (collagen and hyaluronic acid). During construction of the cell layer on the scaffold, the lumen environments are controlled by lumen fluid and gas concentration, among other parameters. In this regard, research is being conducted to construct a model in the form of artificial tissue by fabricating a tubular scaffold. For example, in one study, a group constructed a tube with an inner diameter of 2 mm based on silk proteins and inoculated cells with culturing for up to 8 weeks (Yin et al., 2016). Additionally, various techniques for scaffold fabrication are being developed, such as 3D printing, and many researchers are designing their own tubular culture product by applying several materials and structures.

The advantage of this type of model is that it is easy to create and maintain an anaerobic environment on the lumen side because it the culture environment on the inside can be separated from the outside of the tube using the intestine structure itself. In addition, compared to other models (microfluidic chip and organoid), it can provide high productivity with reproducibility, a larger tissue area, and a longer culture period, successfully maintaining tissue structure. In addition, it is possible to simulate the flow in the gut along the tube, so it could be used in new experiments or even transplants.

For example, studies indicate and demonstrate various perspectives on the tissue-engineered model at various scales. A 3D porous silk protein scaffold, including an engineered hollow lumen structure, was constructed (Chen et al., 2015). The hollow lumen of the 3D scaffold was a secure region to inoculate Caco-2 and HT29-MTX cells. At the same time, primary human intestinal myofibroblasts (H-InMyoFibs) were cultured in the porous bulk space, which was embedded in collagen gel. This culture product, including scaffold and cells, induced typical physiological responses based on the tubular architecture and derived features, with a low oxygen level in the lumen. The results showed secretion and accumulation of mucous substrates on the apical epithelium of the lumen, enabling the *in vitro* model to mimic the interaction with the gut microbiome. Moreover, this 3D model demonstrated its robustness in allowing the tissue structure, activity, and cellular phenotype to be maintained over several months.

In other studies, PLGA scaffolds were utilized by Costello et al. (2014) and a novel designed tubular scaffold was used by Shaffiey et al. (2016) to evaluate the activity of intestine-derived cells, such as cellular growth and differentiation. Recently, researchers expanded the scope to test cellular responses *in vitro* and *in vivo* using intestinal stem cells (with engraftment in animal models). They reported that cells differentiated from scaffolds into crypt–villus structures, and their colonization was enhanced by co-culture with myofibroblasts, macrophages, and the gut microbiome. Remarkably, the implanted scaffolds enhanced mucosal regeneration *in vivo*.

Despite these advantages, there are a few disadvantages. It is difficult to proceed with a complete culture and it is almost impossible to observe the culture directly using a microscope due to the thick layer. Similar to a microfluidic chip, the model requires dedicated equipment. Moreover, the model size and area are larger than those of conventional cultures, so there are some difficulties in simulating and representing the intestinal microstructure uniformly throughout the entire culture product. The tissue model usually simulates the dimension of the intestinal cell layer to a high degree, so it requires a thick and dense cell layer. Due to this complexity, adding the surrounding environments of *in vivo* tissue can be relatively difficult compared to other models, such as microvessels, immune cells etc. In addition, when microorganism co-cultures are processed, they could possibly play a role in confining the lumen from external air. Therefore, the required culture period is longer than that of simple cell culture. Nevertheless, while the disadvantages limit expansion of this technique, it is also suitable for studying or mimicking dynamic intestinal tissue with gut microbiome.

## Development Of Disease Models From *in vitro* Models

The intestine is characterized by having the most complex ecosystem and dynamic environmental changes of all other organs in the body. Nutrients and substrates are primarily supplied, and their amounts are always dramatically changing. Therefore, the ecosystem does not maintain a steady state, and it is possible that events such as diarrhea, inflammatory bowel disease, and infectious diseases occur. Such symptoms are quite common, but they can become serious and painful (Maloy and Powrie, 2011). For a long time, researchers have tried to address those problems and design rational disease models for effective experiments.

Of the various diseases, one major target of disease modeling is inflammatory bowel disease, which occurs as a result of disrupted homeostasis and inflammation. Bacterial or viral infection of the digestive tract is known to be an initial trigger of this disease (De Hertogh et al., 2008). An infection of the digestive tract directly interacts with the mucus layer; therefore, the mucus layer, as a factor in model simulations of inflammatory bowel disease or other infectious diseases, is more important here than the ordinary intestinal model. Animal models have usually been constructed and used to study intestinal diseases. For the construction of such models (Dosh et al., 2019), there are various methods for causing defects in the mucus layer of animal intestine, including chemical treatment, mucin-related gene defection, specific disruption of intestinal epithelial cells, and immune cell deformation (Table 2).

**Table 2:** Commonly used IBD mouse models.

Categories	Model examples	Prevalent type of response	Details of barrier defect	References
Chemical induction	Dextran sodium sulfate (DSS) colitis	Epithelial damage	Deficiency of Muc2, main gastrointestinal mucin.	Persše and Cerar, 2012; Chassaing et al., 2014
	2,4,6-trinitrobenzene sulfonic acid (TNBS)	Epithelial damage, immune-mediated	Coupled with intestinal proteins eliciting significant immunologic response, such as Th1 inflammatory response.	Loeuillard et al., 2014
	Oxazolone	Epithelial damage, immune-mediated	Direct destruction of colonic mucosa and association with Th2-type inflammatory response.	Heller et al., 2002; Weigmann and Neurath, 2016
Spontaneous mutation	SAMP1/Yit	Immune-mediated	Spontaneous inflammation of terminal ileum and cecum driven by TH1 response and epithelial barrier defect, but TH2 response may develop at later stages of disease.	Pizarro et al., 2011
	C3H/HeJBir	Immune-mediated	Increased B-cell and T-cell reactivity to antigens of enteric bacterial flora causing colitis.	Elson et al., 2000
	Nuclear factor κB (NF-κB) essential modulator (NEMO) colitis	Cytokine release	Reduced paneth cell numbers and increased IEC apoptosis.	Liu et al., 2017
Adoptive T-cell transfer	Systemic T-cell activation	Immune-mediated	Cytokine release (TNF, LIGHT) causing MLCK activation and occludin endocytosis.	Chen and Sundrud, 2016
	CD4 + CD45RBhi	Immune-mediated	CD4+ cells from diseased mice displayed highly polarized Th1 pattern of cytokine synthesis.	Byrne et al., 2005
Genetic engineering	IL-10−/− knockout	Cytokine release, epithelial damage	IL-10 signaling in macrophages and neutrophils is necessary to prevent abnormal regulation of responses to normal microflora.	Scheinin et al., 2003
	FOXP3 mutation	Immune-mediated	Autoimmune enteropathy by excessive T-cell activation.	Bamidele et al., 2018
	Dominant negative N-cadherin transgene expression	Epithelial damage	Defective epithelial maturation, migration, and adherens junctions.	Radice, 2013
	MDR1A-deficient mice	Epithelial damage	Reduced occludin phosphorylation, increased epithelial cell response to LPS.	Wilk et al., 2005
	Constitutively active MLCK transgene expression	Epithelial damage	MLC hyperphosphorylation, barrier dysregulation.	Cunningham and Turner, 2012
	JAM-A-deficient mice	Epithelial damage	Effect on epithelial permeability.	Laukoetter et al., 2007
	Mucin-2-deficient mice	Epithelial damage	Intercellular junction defects, mitochondrial damage, and ATP depletion.	Borisova et al., 2020
Microbiome induction	Enteropathogenic *Escherichia coli* infection	Immune-mediated	Type III secretion (of bacterial proteins), MLCK activation, and occludin endocytosis.	Glotfelty and Hecht, 2012
	*Clostridium difficile*-induced colitis	Epithelial damage	Actomyosin disruption and glucosylation of Rho proteins, loss of ZO1 and ZO2.	Best et al., 2012
	Enteric microbial transfer to germ-free IL-10−/− mice	Immune-mediated	Resident enteric bacteria are necessary for the development of spontaneous colitis and immune system activation in IL-10-deficient mice.	Keubler et al., 2015

However, these methods also have limitations, including in terms of their reproducibility, observation scope, cost, and level of experimental difficulty. For direct and reliable experiments, a cell culture model with a mucus glycan layer was developed. In the intestinal model, glycan is added to the mucus layer *via* a mucus-secreting cell line [HT29 methotrexate (MTX) cells, LS174T] or goblet cells. For example, HT-29 MTX was cultured with Caco-2 in various ratios (1:9–3:7) or stem cells were differentiated into several intestinal epithelial cell types (enterocytes, goblet cells, and enteroendocrine cells) to mimic the intestinal and colonic mucosa (Dosh et al., 2019). To create a disease model from an *in vitro* model, various methods are used. One is direct deformation of the mucus layer in the model. Before pathogen treatment, the defensive glycan-rich barrier is removed by a chemical or enzymatic method. Then, researchers can create an obvious disease physiology and observe the interaction and effect of the pathogen in the condition. Another method is to treat with the pathogen over a long time and elicit an inflammatory response from a model with normal physiology and a mucus layer, which can mimic the chronic disease physiology (Zheng et al., 2020).

The simulation degree of this model can be enhanced by increasing the model complexity. In a more complex model, immune cells can be treated, and it is possible to observe immune responses against infectious pathogens. With the use of such a model, immune signaling molecules expressed by infectious stimulation and inflammation were observed. This model can be used effectively to assay the effect of a pathogen or infectious microbe under normal intestinal conditions (Sekirov and Finlay, 2009; Kho and Lal, 2018). The use of gene-modified cells can create a model that more simply simulates congenital disease.

The intestinal culture models introduced in this review for modeling inflammatory or infectious conditions have distinct advantages. First, the cell type (normal cells, transformed cell lines, stem cells, or isolated cells) with the least limitations on physiological conditions can be selected. Many studies have used various cell types for their own purposes. For example, Caco-2 and HT-29 cell lines have been widely used to construct models simulating intestinal layers based on their immortality and accessibility. However, these immortalized cells have significant differences in their state of differentiation, viability, proliferation, metabolic properties, and immune responses. Consequently, models using immortalized cells may be less representative of the normal colonic epithelium, with high differentiation, which may be a disadvantage in mimicking intestinal disease. To circumvent the disadvantages of the abovementioned models, many studies have shown that a disease model can be organized using cells from biopsy specimens obtained from the intestines of IBD patients. These cells have increased expression of inflammatory cytokines, including IL-1β and TNFα (Yamane and Yamane, 2007).

Second, model cultures are available for direct environmental maintenance and control, such as cellular populations, mucus thickness, etc. Third, the culture conditions allow researchers to create phenotypic and morphologic similarities, including the formation of 3D multilayered epithelial tissue or crypt structures and multi-phenotype cells (enterocytes, goblet cells, or enteroendocrine, and goblet cells). In the latter, *in vitro* models can be modified to inoculate cell types of interest into other systems, for example, immune cells for the immune system or smooth muscle cells and fibroblasts for structural enhancement. Therefore, the complexity of the model can be increased by adding more factors to simulate actual human tissue more closely (Sekirov and Finlay, 2009; Coccia et al., 2012).

Recently, *in vitro* models have experienced rapid development; however, their diversity and specific properties as disease models are insufficient compared to conventional animal disease models that have been developed for a long time. The current intestinal culture models have critical limitations that restrict their application in lab-scale experiments and research. The complex conditions mean that these models cannot easily be used in conventional diagnosis or preclinical trials. The conventional process is already certified by the FDA and is widely used in industries as a suitable high-throughput screening system to search for infectious or therapeutic compounds. It is more difficult than culturing with a simple Transwell or other platforms, so it is emphasized that there may be a need to standardize the model as a novel platform. Several models have bright prospects, with the properties required for an intestinal model, including mucus layer, mucus-secreting cells, and microbe treatment, and further studies are needed to improve their reliability and properties so that they can be used as experimental models after collecting more data and conducting big data analysis to verify their relevance and applicability.

## Conclusion and Future Prospects

In the current review, we highlight the structural and functional features of models intended to replace cell-based animal models containing microbiomes and the potential for a disease model derived from infectious inflammation examined with mucin glycan. The gastrointestinal system, which contains the oral cavity–stomach–gut components, is essential for human activity with regard to energy production and complement supply. Because of its importance, homeostasis is tightly regulated, but is surprisingly easy to disrupt, with consequences being diarrhea as well as inflammatory and infectious diseases. For several reasons, food is continuously supplied to the system, whose microbiome is always changing; therefore, pathogens or sources of infection can occasionally invade the system *via* food consumption. The most important underlying reason for this variation is the complexity of the intestinal environment. Even when the same food is supplied, infectious substrates can be produced by microorganisms depending on the conditions in the intestine. The complexity of the intestine is derived from the intestinal tissue and microorganisms. Therefore, it is difficult to construct a rational experimental model, because the structure and environment of the intestine different from those of other tissues. In this context, the model should strike a balance between physiological complexity and experimental simplicity. As a result, from the simplest 2D culture model, various technologies, such as 3D scaffolds, Transwell, microfluidic chips, organoids, and 3D printers, are being used for model construction. With the development of these technologies, the ability to implement physiological complexity in a model that can confirm the interactions between intestinal tissues and microbes is also increasing. Such techniques are applied to create models, with *in vitro* disease models being developed that focus on the glycan-rich layer that protects the tissue from infectious pathogens. The mucin glycan is added to the model by inoculating mucus-secreting cells or differentiating stem cells. In conclusion, novel engineered human intestinal tissue systems that recapitulate normal physiology provide an innovative and attractive approach to modeling inflammatory diseases of the gastrointestinal tract. In future models, a critical issue will be how to ensure uniformity and ease of use while increasing the physiological complexity. Models could provide not only normal physiology but also inflammatory and infectious diseases of the gastrointestinal tract as an attractive approach. Therefore, researchers are focusing on developing a novel platform and standards covering diverse physiological conditions for more reliable data.

## Author Contributions

S-MJ and SK conceived and designed the review manuscript. S-MJ wrote the manuscript draft. SK evaluated and approved the manuscript. All authors contributed to the article and approved the submitted version.

## Funding

This work was supported by the basic science research program (NRF-2021R1A2C1005811) to SK, the National Research Council of Science & Technology (NST)-Korea Research Institute of Bioscience and Biotechnology (KRIBB) postdoctoral fellowship program for Young Scientists at KRIBB to S-MJ. It was also partially funded by Korea Institute of Planning and Evaluation for Technology in Food, Agriculture, Forestry and Fisheries (IPET) supported by Ministry of Agriculture, Food and Rural Affairs (1545021966) and KRIBB Research Initiative Program grant.

## Conflict of Interest

The authors declare that the research was conducted in the absence of any commercial or financial relationships that could be construed as a potential conflict of interest.

## Publisher’s Note

All claims expressed in this article are solely those of the authors and do not necessarily represent those of their affiliated organizations, or those of the publisher, the editors and the reviewers. Any product that may be evaluated in this article, or claim that may be made by its manufacturer, is not guaranteed or endorsed by the publisher.
